# Development of a Content Programming Tool for School-Based Mental Health Literacy Interventions for Adolescents

**DOI:** 10.62641/aep.2403

**Published:** 2026-10-15

**Authors:** Fernando Lezcano Barbero, Alba Herranz Isabel, Rebeca Zabaleta González

**Affiliations:** ^1^Department of Education Sciences, Universidad de Burgos, 09001 Burgos, Spain

**Keywords:** mental health literacy, adolescent, intervention, education

## Abstract

**Background:**

Interest in adolescent mental health (MH) is gaining significant momentum, supported by numerous internationally recognized organizations. Within the educational domain, initiatives have been proposed to enhance mental health literacy (MHL); however, there remains a lack of consensus regarding the optimal sequencing of topics within such interventions. This study aims to identify the essential content to be incorporated in MHL training programs and to establish a structured framework for their delivery.

**Methods:**

A scoping review was conducted in accordance with Preferred Reporting Items for Systematic Reviews and Meta-Analyses extension for Scoping Reviews (PRISMA-ScR) guidelines by three independent researchers. The review included reports detailing school-based MHL interventions targeting adolescents. An initial search yielded 470 reports, from which 26 articles met the inclusion criteria following systematic protocol development. Data extraction focused on identifying the MHL-related content addressed across the analyzed programs. Utilizing an inductive approach, findings were synthesized by coding information into thematic categories.

**Results:**

Analysis revealed fourteen thematic categories. The primary knowledge and skills addressed encompassed the concept of MH and mental health problems (MHPs), stigma reduction, help-seeking, lived experiences, early intervention and prevention, promotion of wellness, self help techniques, and aid skills. Notably, few programs incorporated direct contact with individuals possessing experience of MH conditions.

**Conclusions:**

MHL programs targeting adolescents should commence with the dissemination of foundational knowledge, followed by stigma reduction facilitated through authentic narratives and promotion of help-seeking. Subsequently, awareness of early intervention and prevention should be emphasized, alongside the development of protective behaviours, coping strategies and communication skills. Finally, interventions should work on MH crisis recognition and first aid competencies. This proposed framework culminates in an educational programming tool conceptualized as a hierarchical pyramid. It is recommended that individuals with experience in MH challenges, who have undergone recovery and received appropriate training, be actively involved in the design and implementation of these programs. Future MHL programs are encouraged to adopt this framework and rigorously evaluate its effectiveness.

## Introduction

Mental health (MH) is considered a fundamental human right, an essential element for personal and community development, and a key aspect of public health [1, 2]. It is defined as a state of wellbeing in which a person can effectively perform daily activities, manage routine stressors, work productively, and contribute to their community [3].

One in seven people between the ages of 10 and 19 lives with a mental health problem (MHP) [4]. Adolescence represents a critical stage for MH, as the majority of emotional disorders manifest between the ages of fourteen and eighteen [5]. The third objective of the Comprehensive Mental Health Action Plan 2013–2030 by the World Health Organization [2], advocates not only for meeting the needs of already diagnosed individuals, but also for promoting initiatives focused on prevention, early intervention, and the promotion of MH, with particular attention to the adolescent population sector.

It is essential that children and adolescents acquire the knowledge and skills necessary to care for and maintain their emotional wellbeing, seek and provide appropriate support, and respond effectively to environmental problems [5]. Deficits in MH knowledge, coupled with stigmatizing attitudes, can worsen symptoms, hinder help-seeking, and adversely affect overall wellbeing and development [1, 6, 7].

Health Literacy refers to the knowledge and social skills which determine the motivation and ability to promote and maintain good health [7, 8]. These competences serve as a protective factor for wellbeing that can be developed through educational strategies [8].

Mental health literacy (MHL) arose from the field of health literacy [7, 8], and refers to the set of knowledge and beliefs associated with MHPs which enable their recognition, management, and prevention [9]. This concept has evolved in the field of public MH, and includes four components: (1) understanding how to obtain and maintain emotional wellbeing, (2) understanding MHPs and their treatment, (3) reducing stigma toward MHPs and (4) promoting help-seeking [8]. This updated definition contemplates three interrelated elements: knowledge, attitudes, and help-seeking efficacy [10], and shifts the focus from a disease-oriented approach to a perspective that considers aspects related to positive MHL [5].

Given the significant amount of time that adolescents spend in educational settings, schools provide an optimal context for implementing initiatives aimed at enhancing MHL [6, 11]. Weist *et al*. [7] emphasize that, considering adolescents’ emotional vulnerabilities, integrating MHL training in schools is increasingly imperative. Evidence suggest that such interventions in educational contexts are associated with improvements in MH knowledge, help-seeking behaviours, social skills, and attitudes towards people with MH experiences [7, 12]. However, the available evidence only allows the effectiveness of these programs to be considered as promising, since the substantial heterogeneity of interventions hinder the formulation of robust conclusions [13].

Recent reviews [11, 13] indicate considerable variability across programs regarding content, implementation and structure. These inconsistencies have been identified as a significant gap within school-based MHL research [13], as the absence of a standardized curricular framework undermines the capacity to assess program effectiveness and delineate best practices [6, 13]. Accordingly, the literature highlights the pronounced need to harmonise MHL initiatives through consensus on a clearly defined content curriculum [6, 13].

Furthermore, these school-based interventions should be pedagogically familiar to students and to the agents responsible for implementing them [6]; therefore, it is appropriate to provide evidence-based MHL curriculum guidelines that support education professionals in planning and implementing such initiatives.

These resources could help ensure that the educational approach to MH goes beyond punctual, less effective interventions [6], and instead is operationalized through sustainable actions tailored to the specific context. Besides, enhancing MHL from early stages could lead to improved emotional wellbeing outcomes [10], and, consequently, alleviate the growing demand for MH professionals and resources.

In light of these considerations, a pertinent inquiry arises: which topics should adolescent-focused MHL educational programs encompass, and how should their content be systematically structured? This study aims to address this question by delineating the essential content components, and proposing a framework for the organization of school-based MHL curricula targeting adolescents.

## Methods

Given the heterogeneity that characterizes the object of study, a scoping review was carried out following the guidelines established by the Preferred Reporting Items for Systematic Reviews and Meta-Analyses extension for Scoping Reviews (PRISMA-ScR) guide [14].

In line with the research question posed, and following the Participants-Concept-Context (PCC) strategy [15], the following inclusion criteria were defined:

- Participants: Publications describing interventions targeting adolescents aged ten to nineteen years.
- Concepts: Quasi-experimental studies assessing a MHL program and providing specific information related to the content addressed.
- Context: Studies conducted in the educational setting.

In order to focus on primary research studies, documents from the gray literature, research protocols, doctoral dissertations, and systematic reviews were excluded. The resources available only allowed to consult Open Access articles.

The information sources consulted were Web of Science and Scopus. All articles published in English and Spanish through February 2025 were considered.

To meet the inclusion criteria, the Boolean phrase defined was: (Mental health literacy AND (Intervention OR Program) AND School AND Students).

The selection process consisted of two review phases: (1) reading of the title and abstract and (2) review of the full text. All authors participated in the review, disagreements were solved by consensus.

The search equation yielded a total of 470 documents. After removing duplicates (59), closed-access documents (264), systematic reviews (22) and a doctoral thesis, 124 articles were obtained. In an initial stage based on reading the titles and abstracts of the articles, 18 publications were excluded for not presenting results and 61 were discarded for not meeting the inclusion criteria. After a detailed review of the remaining documents, 5 articles were excluded for not addressing MHL, 3 for not being interventions implemented in the educational setting, 2 for not providing a description of the program, 1 for not presenting results, 6 for not measuring aspects related to MHL, 1 for being a descriptive study, and 1 for not meeting the participant-related inclusion criteria. The systematic development of the search and review protocol yielded a total of 26 valid articles.

In Fig. 1, following the guidelines established by the Preferred Reporting Items for Systematic Reviews and Meta-Analyses (PRISMA) 2020 statement [16], the flow diagram of the process of identification, screening, selection, and inclusion of valid articles for synthesis is presented.

**Fig. 1.**
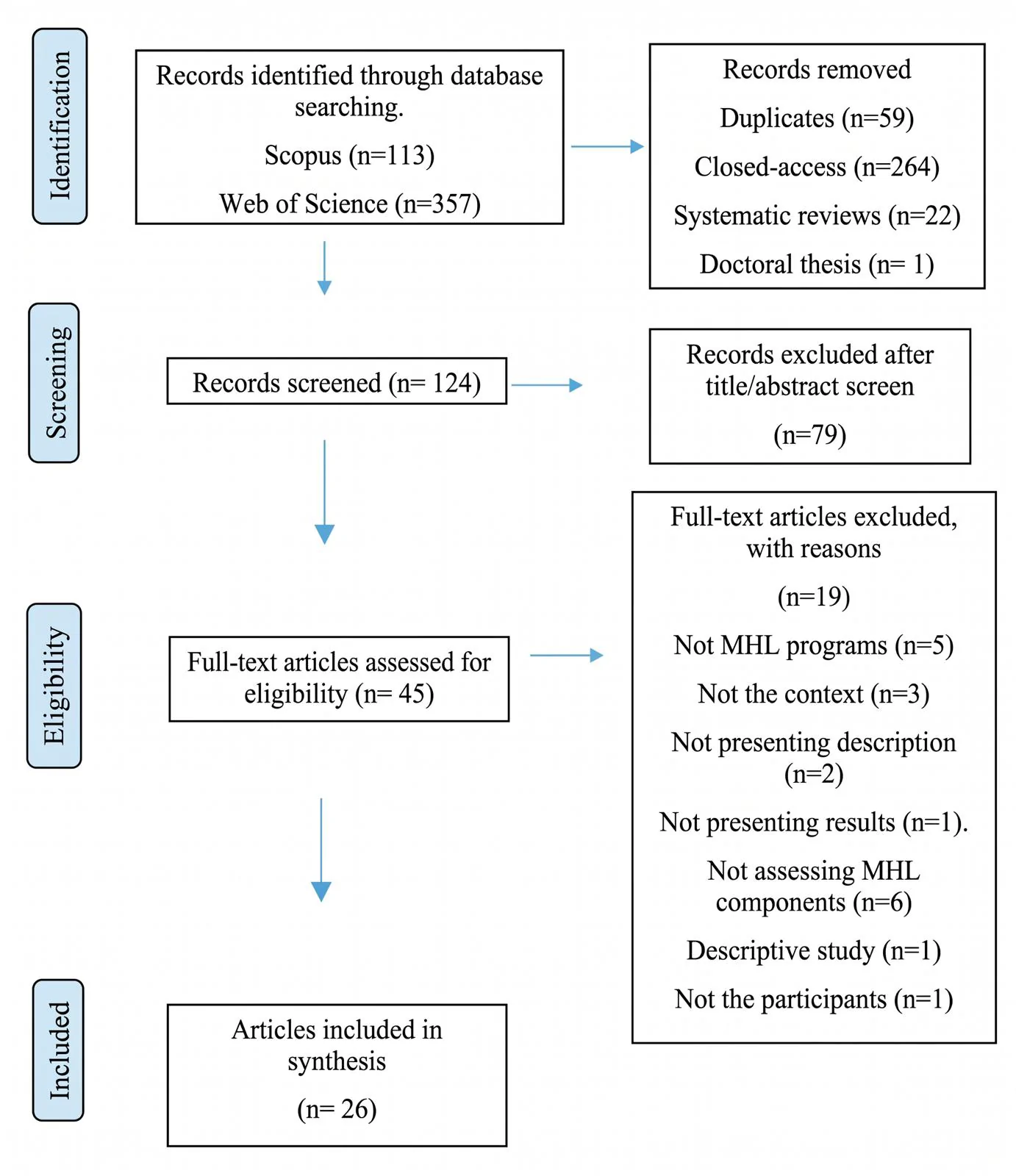
**Article search and selection process—PRISMA flowchart**. Based on Page *et al*. [16], prepared by authors. Description of the process adopted to identify, select, determine eligibility, and include studies in the review. MHL, mental health literacy.

To meet the study’s objective, data extraction focused identifying the MHL content addressed within each analyzed program. The selected studies’ data were systematically charted using a standardized tabular tool. Independently, two authors completed this table by documenting the content identified in each study. Upon comprehensive review, the authors, through an inductive approach, synthesized the data by coding the information into different thematic categories. After performing coding independently, disagreements were solved through consensus with the third researcher. Finally, the frequency with which these categories appeared was quantified.

## Findings

Table 1 synthesizes the content covered in the reviewed studies, outlining the knowledge and skills incorporated in the programs, ranked according to frequency of inclusion (second column).

**Table 1.** **Summary of content addressed in programs**.

Content	No. programs
MH concept.	23
MHP concept.	23
Specific MHPs.	22
Help-seeking.	20
False myths related to MHPs.	16
Real experiences of MHPs.	15
Protective behaviours.	13
Emotional management tools.	10
Treatment of a MHP.	10
MH support skills.	5
Resilience and recovery.	5
Prevention and early intervention.	4
Emotional communication.	3
MH crisis.	3

The first column outlines the main topics addressed in school-based MHL interventions. The second column displays the frequencies of these topics. MH, mental health; MHP, mental health problem.

To allow a fluent reading, most citations presented in this section are merely illustrative.

The reviewed programs addressed a wide variety of topics related to MHL. With the exception of the proposals by Skre *et al*. [17], Chisholm *et al*. [18], and Lai *et al*. [19], all the programs addressed the concepts of MH and MHPs, as well as their characteristics, and risk factors.

Twenty-two programs treated specific MHPs such as depression [20], anxiety [21], or eating disorders [22], among others. They discussed their signs, causes or risk factors, and treatment.

Frequently, the programs addressed help-seeking [20, 23, 24], providing information on available resources, how to access them and potential barriers. In addition, sixteen proposals [18, 20, 23] discussed false myths related to MHPs, and fifteen presented real-life experiences either in person [18, 20, 25], virtually [24, 26, 27, 28], or combining both formats [23, 29, 30]. Publications by Kutcher *et al*. [31], Nguyen *et al*. [32], Mcluckie *et al*. [33], Simkiss *et al*. [34], and Yang *et al*. [35] do not specify how the testimonials were presented. With exception of the study by Casañas *et al*. [20], all the proposals provided training to these individuals before their intervention. However, none of the articles provide information about the specific training provided.

Other common topics were protective behaviours for emotional wellbeing (such as healthy lifestyle and positive behaviours) [20, 22, 24], emotional management strategies (i.e., stress coping skills, dealing with adversity or problem solving) [26, 27, 36], and the treatment process (main treatments available, therapies and medication) [24, 25, 37].

Five programs addressed skills to support the MH of others [22, 38, 39, 40] as well as resilience and the possibility of recovery [24, 25, 26, 27]. Four proposals discussed the importance of prevention and early intervention [22, 24, 38, 39].

Finally, three programs worked on communication skills [19, 26, 29] and the management of MH crises in oneself and others [38, 39, 41].

## Discussion

In alignment to the findings of Zachik *et al*. [13], the present study identified a broad spectrum of topics incorporated within the analyzed programs.

These results are congruent with the developmentally appropriate contents proposed by Kågström *et al*. [5] for working on MHL with people aged twelve to eighteen. This suggests that such knowledge and skills should be integrated in the design of prospective educational interventions for adolescents.

Most proposals addressed the concepts of MH and MHPs, as well as their signs and risk factors. Given that recognizing the importance of emotional wellbeing relies subject-specific knowledge [42], it is advisable to disseminate information regarding MH and related disorders. Moreover, knowledge acquisition facilitates the processing of complex information and fosters a deep understanding, thereby strengthening overall MHL [43]. Accordingly, educational programs should commence with the provision of factual information about MH.

Programs often provided information about specific MHPs. Interventions may either work on general MHL or focus on specific diagnoses targeting adolescents identified as at risk [5]. Considering that actions should be tailored to the characteristics and context of the participants [8], the decision to include information on specific diagnoses should be informed by the students’ needs and school circumstances.

Once factual knowledge is acquired, adolescents tend to exhibit reduced stigmatizing attitudes toward MH [44]. Adolescents are particularly vulnerable to stigma due to their heightened need for social acceptance and peer influence [45]. A qualitative study [46] demonstrated that negative perceptions of MH issues hinder their acceptance and disclosure, as well as subsequent help-seeking, underscoring the importance on stigma reduction efforts subsequent to the delivery of factual information [44].

Despite prior research has indicated that exposure to lived experiences related to MH can reduce stigma and facilitate the acquisition of knowledge related to MHPs [42], the program conducted by Chisholm *et al*. [18] reported that contact does not significantly influence MHL. These results could be influenced by the omission of an information-provision component in the intervention, given that previous studies [44] highlighted the need to have acquired certain prior knowledge to correctly process testimonials. In light of these findings, it is recommended that real-life experiences be incorporated in MHL programs only after participants have attained sufficient knowledge to effectively process such information [47].

Knowledge about MH and positive beliefs and attitudes towards MH conditions notably influences help-seeking behaviours [46, 48]. Knowing this, after providing information and working on stigma and real experiences, it is appropriate to work on available help resources and treatment options in MHL interventions [5]. Considering the young age of the participants, it is also advisable to disseminate knowledge and skills related to disclosing the problem to trusted adults [5].

In line with the findings from previous reviews [6], we found that few programs addressed the promotion and maintenance of MH, reflecting an approach focused primarily on illness rather than on strengthening wellbeing. The updated conceptualization of MHL extends beyond a comprehensive and non-stigmatizing understanding of MH, and the available support resources, to include the ability to obtain and maintain mental equilibrium [8]. To align with this more salutogenic perspective, future programs should concentrate on protective behaviours (such as healthy lifestyle and social engagement) [6] after first addressing on recognition, knowledge and beliefs.

Given that adolescence is a key period in which sustainable and long-lasting habits are acquired [5], the enhancement of positive behaviours in this stage would benefit the MH of future citizens at both individual and societal level [10].

The analyzed programs also included aspects related to emotional regulation. Coping strategies are adaptive responses oriented to reduce environmental demands [49]. As adolescents increasingly attain independence from their families and encounter more complex cognitive, emotional, and social demands [50], it is important for them to acquire a range of functional coping strategies to respond adaptively. To facilitate the development of strategies that support the maintenance of MH, MHL interventions should disseminate self-care tools such as positive self-talk or relaxation techniques [5].

Some proposals included content related to support skills. Empirical evidence indicates that adolescents preferentially seek emotional support from close friends [51], and value peer advice when disclosing MH concerns or seeking help [52]. MH first aid is defined as assistance provided to individuals developing MHP or experiencing crises [53]. Building knowledge and competencies on how to support to their peers represents a promising approach to enhance MH of adolescents [53]. The Teen Mental Health First Aid program, developed by experts and people with experience, focuses on crisis recognition and first aid skill development [53, 54]. This intervention has demonstrated substantial efficacy in fostering help giving intentions and behaviours [55]. Its curriculum initiates with MH knowledge, stigma reduction, help sources and self help before progressing to crisis recognition and first aid techniques [53, 54]. This suggests that MHL interventions may address crisis recognition and aid skills once knowledge, stigma, help-seeking, individual protective factors and coping strategies are covered.

Certain interventions introduced resilience and the possibility of recovery from MHPs. The recovery paradigm emerged during the deinstitutionalization of psychiatric service users, has shifted MH care focus from symptom reduction toward fostering social connectedness, hope, positive self-perception, life purposes, and empowerment [56, 57]. Authors such as Guerrero *et al*. [56], advocate for addressing MH interventions through this approach, so MHL programs should embrace and disseminate this conceptualization of recovery. This recovery-oriented perspective can be operationalized within interventions by presenting authentic recovery narratives delivered by individuals with live experience of MH conditions. Peer Support Workers—young individuals who have experienced MH challenges, and achieved substantial recovery-provide emotional support to other young people following appropriate training [58]. Given their role as exemplars of recovery [58], future research may involve these professionals in the design and implementation of MHL programs and assess the effect of their participation.

Certain initiatives emphasize the critical importance of prevention and early intervention in MH care. These strategies are widely recognized as fundamental to mitigating the potential adverse impact of emotional disorders, thereby underscoring the necessity of incorporating youth-focused health-care approaches centered on prevention and early intervention [59]. Accordingly, it is essential to raise adolescent’ awareness of these concepts within MHL programs. Recent systematic reviews [60, 61] indicate that difficulties in symptom recognition, negative attitudes toward MH and insufficient knowledge of available resources impede timely access to treatment. Consequently, it is advisable to incorporate content on early intervention awareness subsequent to foundational coverage of knowledge acquisition, stigma reduction, and help-seeking intentions. Given that cultivating skills associated with emotional wellbeing constitutes an effective preventive strategy for adolescents [62], MHL programs should prioritize enhancing preventive awareness prior advancing emotional protection and manage competencies.

Although the development of emotional communication skills was infrequently addressed in the programs, studies highlight its critical role in emotional regulation [63], and in fostering and sustaining healthy interpersonal relationships [64]. Emotional expression, defined as the articulation of internal emotional states, facilitates engagement with one’s feelings and management of complex emotional processes [65]. Based on this rationale, MHL interventions are recommended to incorporate training on emotional expression skills alongside emotional coping strategies. Furthermore, communication skills contribute to the prevention and resolution of interpersonal conflicts, promote empathetic understanding of others’ perspectives and emotions, and support the establishment of meaningful relationships [64]. These competences have been recognized as fundamental to the development of support skills [54], so programs may consider introducing them prior to first aid training.

The cognition preceding affect model in health behavior posits that ideas elicit emotional responses, which subsequently motivate health-related behaviours [66]. In alignment with this framework, MHL interventions should commence with the dissemination of knowledge, followed by efforts change beliefs and attitudes, culminating in the promotion of behaviours that safeguard one’s own emotional wellbeing as well as that of others. It is imperative the program designs be tailored to accommodate the developmental stages and specific needs of participants, while also accounting for their socio-cultural contexts [5, 8].

After comparing the findings of this study with the available evidence, and using the cognition preceding affect model as a reference, Fig. 2 presents a standardized curriculum guide for sequencing the content of MHL school-based interventions.

**Fig. 2.**
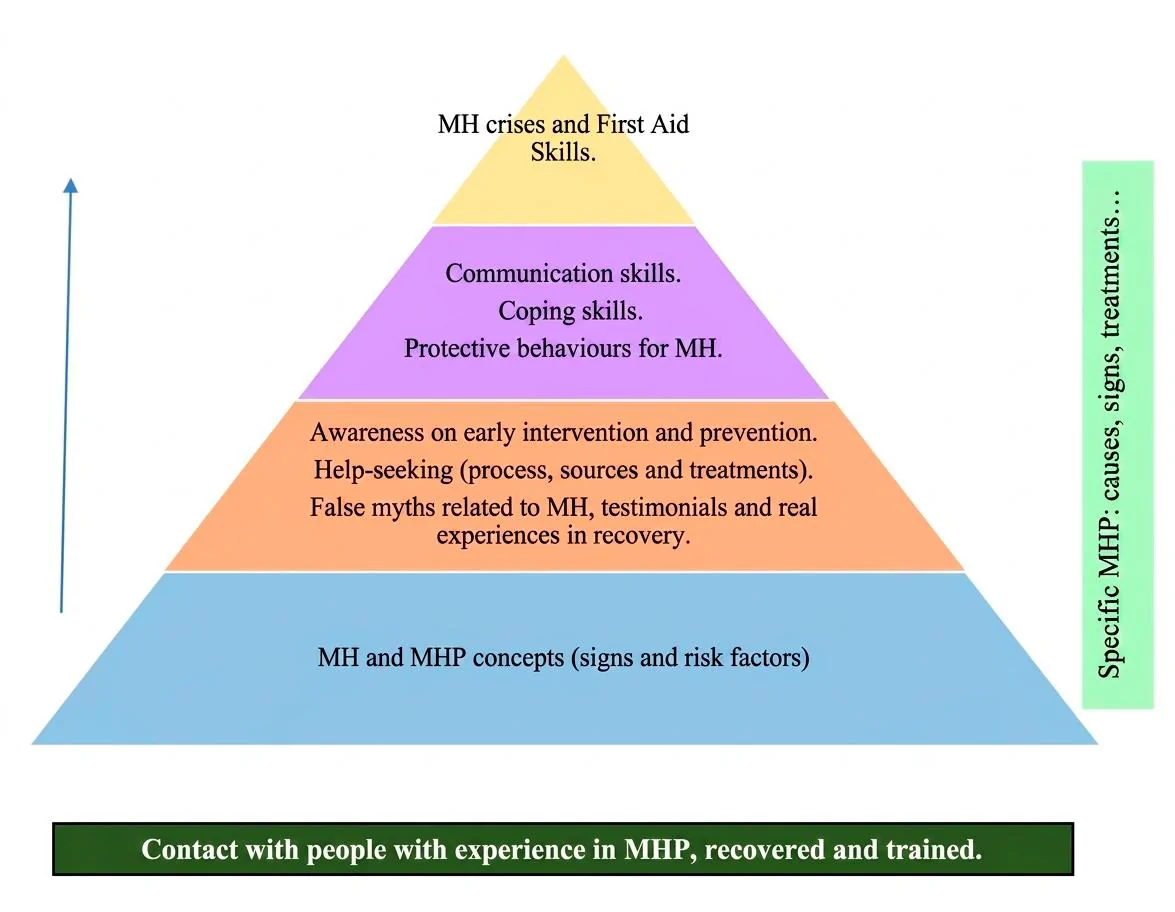
**MHL school-based interventions programming tool**. This graphic tool presents a progressive curricular structure of MHL content to facilitate the design of MHL interventions for adolescents. MH, mental health; MHP, mental health problem. Prepared by authors.

Finally, few proposals incorporated direct contact with individuals possessing personal experience in MH. Intergroup contact theory posits that interaction between distinct groups can mitigate prejudice and discrimination [67]. Moreover, it has been recognized that people with MH experience hold unique and valuable insights into MH due to their backgrounds [42]. Accordingly, relevant entities such as the World Health Organization [2], and scholars as Sexsmith Chadwick *et al*. [68], advocate for involvement of people with lived experience in MH in initiatives aimed at promoting adolescent emotional wellbeing.

Consequently, some educational interventions engage people with lived experience of MHPs to share their narratives with participants [18]. The introduction of contact in combination with education has been recognized as a promising strategy to enhance MHL [69].

However, recent reviews [47] indicate that the application of this model leads to divergent and even contradictory conclusions; therefore, further studies are needed that apply this strategy and rigorously evaluate its effectiveness.

None of the reviewed articles detailed the specific training provided to individuals with lived experience prior to their involvement in interventions. Given that recent reviews [1] emphasize the critical importance of adequately preparing all stakeholders engaged in MHL proposals, future research should focus on developing and validating training programs to equip people with experience in MH to effectively contribute to the promotion of MHL within educational settings.

In line with the statements of Guimón [70], a key figure in the psychiatric field, only the compromise of educators will make it possible to attend child and adolescent MH within the school context. Looking ahead, it will be important to establish collaborative networks between education and MH professionals, as well as people with lived experience of MH, to enhance MHL of the future citizens [1].

Although this study adhered to PRISMA-ScR standard guidelines and involved three independent researchers to ensure methodological rigor, this study is not without limitations. First, certain information may have been excluded due to its presence solely in gray literature or has not been published in the selected databases. Second, language restrictions potentially limited the inclusion of pertinent studies. Additionally, reliance on published articles may have resulted in the omission of programmatic details not reported in papers. Finally, as inherent to scoping review methodology, quality of the included documents was not assessed.

## Conclusions

This study aimed to delineate the essential content and structural organization of MHL interventions for adolescents. The research question was addressed by contrasting results with the evidence available.

Based on the findings, a sequential framework for MHL content delivery is proposed. Interventions for adolescents should commence with the provision of factual information concerning MH, including definitions, symptomatology and risk factors. Subsequently, stigma reduction should be addressed, complemented by the presentation of authentic testimonials reflecting lived experiences and recovery. Following this, proposals should introduce help-seeking information by outlining available resources and treatments, alongside fostering positive attitudes toward support seeking. Awareness of prevention and early intervention should be incorporated thereafter. Subsequently, actions should cultivate individual competencies related to protective behaviours, coping mechanisms and communication skills. Finally, programs should focus on MH crisis recognition and first aid training.

To ensure contextual relevance, program developers are encouraged to tailor content to specific disorders based on the needs of the student population.

It is advisable to involve young individuals with lived MH experience in the design and implementation of MHL initiatives for adolescents; these contributors should receive specialized training prior to participation.

The present study contributes to the body of knowledge related to school-based MH by proposing an evidence-based didactic planning tool. This contribution supports the standardization of MHL initiatives and facilitates the identification of best practices to collaborate with the promotion of adolescent mental wellbeing.

To support the appropriate integration of MH into the educative field, it is crucial to strengthen educators’ commitment and to establish collaborative networks with MH specialists.

Future research should empirically validate this framework. Moreover, the development of specialized training programs for individuals with MH experience engaged in educational roles, as well as the evaluation of their impact on adolescents’ MH promotion efforts, represent a valuable direction for subsequent inquiry.

## Availability of Data and Materials

Main data generated or analyzed are included in the manuscript. To access the study data and materials that support results and analysis, contact the corresponding author.
